# Some Diseases of the Ourang-Outang*From “Il Medico Veterinario.

**Published:** 1885-07

**Authors:** R. Bassi


### II.—SOME DISEASES OF THE OURANG-OUTANG.*

BY PROF. R. BASSI.

*Acute Intestinal Catarrh.*—Case 1. A male ourang-outang, aged about five years, that had been imported to Italy about a year and a-half previous, was placed in the Zoölogical Gardens of Turin. This animal, while in the hands of its former owner, had suffered from a severe illness, of the nature of which I hvae not been able to obtain exact information, and in consequence of this disease it had lost all its hair, but had entirely recovered from the attack, and afterwards increased in flesh. For about two months after its entrance into the Garden, with the exception of four days, during which he was attacked with rheumatic fever of moderate intensity, the animal was quite well. Then he presented *l'herpes labialis* of the identical form as that which is not so rarely observed in the human species after accession of intermittant fever. After this time the animal appeared weak and melancholy. It had intense fever, frequent discharges from the bowels, copious and liquid, of a yellowish color. These discharges occurred sometimes with manifest signs of tenesmus. The acute intestinal catarrh was aggravated progressively until the death of the animal, which took place eight days after the attack. During the last two days of his life he had become so emaciated and so weak that with difficulty he moved on his bed. All through his sickness the animal drank nothing more than a little broth and ate only some pieces of musk melon. The only thing

* From "Il Medico Veterinario, he appeared to enjoy was a little lemonade. This disease was caused by a lowering of the temperature of the atmosphere. I had not the opportunity of making an autopsy.

Case 2. Another ourang-outang, female, was attacked by the same disease. She had frequent discharges from the bowels of a yellowish color, great thirst, no appetite, convulsive movements of the lips and intense fever. I administered simple syrup with a few drops of laudanum during the day. No benefit was derived from this remedy, and the diarrhœa continued nineteen days, at which time the animal died. No autopsy was made.

*Tuberculosis.*—We received from Marseilles an ourang-outang, female, not quite four years old. The animal was in good condition, active, and did not appear to suffer from the journey to the garden, although made in winter, but she coughed occasionally. Early in the spring the animal commenced to lose appetite and to be capricious in the choice of her food. At times she had fever and a dry cough. This state of things continued, with alternations of improvement and relapse for about a month, after which she refused all food until relieved by death. An autopsy was made fifteen hours after death. Animal was well nourished, and the flesh, in color and consistency, similar to that of a chicken. The fat was in great layers, as in the pachyderm, and there appeared also other large masses around the kidneys, spleen, among the folds of the mesentery and of the large omentum. The digestive tube was very pallid along its whole length and distended by gas. Fatty degeneration of the liver existed and there was tuberculosis of both kidneys, but the tubercles were few and small. Large tubercles were found in the spleen, but they existed more abundantly in the lungs, where there was also found serious hepatization, especially in the right one. Tubercles were not softened in any of the organs. The spleen was adherent to the diaphragm. The animal, when purchased certainly had tuberculosis, but there was no indication of it. It would have lived, perhaps, for several months longer, if it was not for an attack of pneumonia, caused probably by some sudden and marked change in the temperature. Some pieces of the intestines invaded by tuberculosis served to practice inoculation in six India swine and a peccary. Inoculation was successful in some of the swine and not in the peccary. Three months after inoculation the swine were killed and the lungs and spleen were found largely invaded by tubercles.

*Paroxysm of an epileptic form.*—A female ourang-outang, which appeared quite well suddenly broke from its keeper, worked in convulsions, gnashed her teeth and foamed at the mouth. This lasted a few moments and the animal in a short time was restored to its usual vivacity. Nothing further was observed by the keeper for another week, when similar symptoms again appeared. Informed of the fact, I carefully examined the animal, and found nothing abnormal, except the tongue was somewhat sore at the apex, which was evidently caused by a bite during the convulsion. Not having succeeded in finding out what caused the epileptic attack, I applied remedies in accordance with the symptoms—hydrate of chloral, after which the epileptic fit did not occur.

